# Factors associated with frailty status compared to pre-frailty in community-dwelling older adults: a cross-sectional study

**DOI:** 10.3389/fpubh.2025.1728208

**Published:** 2026-01-14

**Authors:** Wei Li, Xiaodan Xue, Dandan Li, Yi Pan, Meiqiu Xie, Ying Zhang, Wenlong Zheng, Mingyue Zhang

**Affiliations:** Department of Non-communicable Disease Control and Prevention, Tianjin Centers for Disease Control and Prevention, Tianjin, China

**Keywords:** community-dwelling, cross-sectional study, frailty, pre-frailty, risk factor

## Abstract

**Objective:**

To identify factors distinguishing frail from pre-frail status in community-dwelling older adults and construct a risk prediction model with logistic regression as the primary method to identify independent risk factors, and a neural network as a supplementary approach to explore complex relationships.

**Methods:**

The FRAIL Frailty Screening Scale was used to screen adults aged 65 and above meeting inclusion criteria for pre-frailty and frailty. A cross-sectional survey collected basic information and disease status, while scales assessed nutritional risk, sarcopenia, and activities of daily living (ADL), alongside physical measurements. Binary logistic regression was used as the primary method to identify factors associated with frailty status. A multi-layer perceptron neural network was employed secondarily to explore complex, non-linear associative patterns.

**Results:**

Of 1,451 participants, 46.0% were pre-frailty, and 54.0% were frail. Age, education level, smoking status, number of chronic diseases, SNAQ nutritional risk, SARC-F sarcopenia, and ADL disability were independent associated with frailty status, with SARC-F sarcopenia showing the strongest association with frailty status. The neural network model identified SARC-F sarcopenia (100.0%), waist circumference (67.0%), and age (62.8%) as the most influential factors.

**Conclusion:**

Our findings identify several factors strongly associated with frailty status in a cross-sectional sample. While these factors represent potential targets for intervention, future longitudinal studies are needed to confirm their predictive value for frailty progression. The logistic regression model provides clinically interpretable factors associated with frailty (e.g., SARC-F sarcopenia, ADL disability, nutritional risk). The neural network analysis corroborated the paramount importance of sarcopenia and highlighted additional non-linear associations. Community decisions should be primarily based on the interpretable outputs of the logistic regression model. Given the cross-sectional design, these findings represent associations at a single time point.

## 1 Introduction

The global aging process is accelerating. According to the World Health Organization, the population aged 65 and above is projected to reach 2 billion by 2050 (1). This global trend is particularly pronounced in China, where the rapid increase in the older adult population presents substantial public health challenges. As of 2021, the population aged 60 and above was approximately 260 million (18.70%), while those aged 65 and above numbered about 190 million (13.50%). Projections suggest that the older population will reach 400 million by 2033 (2, 3). A critical consequence of this demographic shift is the rising prevalence of frailty, a clinical state of heightened vulnerability characterized by diminished physiological reserves and reduced resistance to stressors (4). A meta-analysis spanning 62 countries reported a frailty prevalence of 24% and a pre-frailty prevalence of 49% among individuals aged 50 and above (5). In Chinese community-dwelling older adults, frailty prevalence varies from 7 to 42% (6).

Frailty is a dynamic, progressive process, representing a critical intermediate state between robust healthy and disability. Research on frailty transitions has gained increasing attention, as it helps identify pivotal intervention points. Frail older adultsare at high risk for adverse outcomes, including falls, disability, prolonged hospitalization, and mortality (7–12). Importantly, the pre-frailty state—a precursor to full frailty—holds significant clinical value. Evidence suggests that early identification and intervention during pre-frailty can delay or even reverse its progression, thereby preventing adverse health events (13–15).

Frailty status is associated with a complex interplay of factors beyond pure pathophysiology. A growing body of longitudinal research highlights the crucial roles of psychosocial determinants (such as social isolation, cognitive decline, and depression), life-course factors (like socioeconomic status), and community-level influences (including the built environment and access to healthcare) (16–18). The multifactorial and non-linear nature of these relationships makes them particularly suited for investigation using advanced analytical techniques. While traditional statistical methods like logistic regression have been widely used to identify risk factors, recent studies have begun to leverage machine learning models, including neural networks, to capture complex interactions and improve classification accuracy for frailty status (19, 20).

Therefore, to address the multifaceted nature of frailty, this study employs a cross-sectional field survey to comprehensively analyze factors associated with frailty status (compared to pre-frailty) in community-dwelling older adults. We aim to apply and compare a conventional logistic regression model with a neural-network-based approach to identify factors associated with frailty status in a cross-sectional design.

### 1.1 Participants

From June to December 2024, 1,451 community-dwelling older adults with pre-frailty or frailty were screened using questionnaires across nine districts in Tianjin City. A convenience sampling method was used to recruit participants from nine districts in Tianjin City. While this approach facilitated data collection, it may limit the representativeness of the sample, as participants from these specific districts may not fully reflect the broader population of community-dwelling older adults. All participants provided informed consent. The study was approved by the Ethics Committee of the Tianjin Center for Disease Control and Prevention (TJCDC-R-2023-019). Inclusion Criteria: (1) Age ≥65 years; (2) FRAIL Scale score of 1–2 (pre-frailty) or ≥3 (frailty); (3) Life expectancy >6 months; (4) Ability to consent to participation. Exclusion Criteria: (1) FRAIL score = 0 (non-frail); (2) Receiving palliative care; (3) Participating in other case management programs (e.g., ongoing frailty interventions from community health centers). Withdrawal Criteria: Participants unable to continue due to sudden events, as determined by the research team.

### 1.2 Data collection methods

This study employed a cross-sectional design, which limits causal inference but allows for the identification of factors associated with frailty status. After obtaining permission from relevant authorities, researchers entered the field, screened participants based on inclusion and exclusion criteria, explained the study’s purpose and significance face-to-face, and obtained signed informed consent. Frailty assessments were performed, and questionnaires were completed by researchers using consistent language, avoiding suggestive phrasing. Each questionnaire took 20–25 min to complete, was distributed and collected on-site, and checked immediately for completeness to ensure data validity.

### 1.3 Questionnaire content

#### 1.3.1 Basic information questionnaire

This questionnaire was developed for this study (Supplementary material 1). Included demographics and health status: gender, age, marital status, education level, smoking and alcohol use, living arrangements, medical payment methods, personal and family income, sleep duration, and number of chronic diseases.

FRAIL scale (21): assessed frailty with five items: fatigue, resistance, ambulation decline, presence of ≥5 diseases (e.g., hypertension, diabetes, cardiovascular events, chronic heart failure, chronic lung disease, arthritis, asthma, laryngitis, stroke, cancer, kidney disease), and weight loss. Each item scored 1 point (total 0–5): 0 = non-frailty, 1–2 = pre-frailty, ≥3 = frailty.

Simplified nutritional appetite questionnaire (SNAQ) (22): a simple, rapid tool with four items (appetite, taste, meal frequency) to assess nutritional risk and dietary intake in the older adults, facilitating nutritional intervention.

SARC-F questionnaire (23): quickly identifies sarcopenia with five items: strength, walking assistance, rising from a chair, stair climbing, and falls. Scores range from 0–10 (0–2 per item); ≥4 predicts sarcopenia and adverse outcomes, with higher scores indicating greater risk.

ADL scale (modified barthel index, MBI) (24): assesses daily living ability (eating, bathing, grooming, dressing, continence, toileting, transfers, walking, and speed). Total score: 100 (fully independent), 60–95 (mild impairment), 40–60 (moderate), 20–40 (severe), <20 (complete dependence).

The internal consistency of the multi-item scales was assessed using Cronbach’s alpha in this study sample. The results showed acceptable reliability, which is consistent with their original validation: SNAQ (*α* = 0.76) (22), SARC-F (α = 0.81) (23), and ADL (α = 0.89) (24). The FRAIL scale, being a simple aggregate of dichotomous clinical indicators, is typically not assessed for internal consistency in this manner, as coefficient alpha is not an appropriate measure for such composite indices (25). Its validity is derived from its widespread clinical use and prior validation studies (21).

### 1.4 Physical measurements

Measurements were conducted by trained investigators using standardized methods. All eligible participants underwent height, weight, waist circumference, arm and calf circumferences, skinfold thickness, blood pressure, grip strength, and body composition assessments. Height was measured with a metal column height meter (accuracy: 0.1 cm), weight with an electronic scale (0.1 kg), circumferences with a tape measure (0.1 cm), skinfold thickness with a caliper (0.1 mm), blood pressure with an electronic monitor (1 mmHg), and grip strength with an electronic dynamometer (0.1 kg). All instruments met national metrology standards, and methods complied with the *Human Health Monitoring Measurement Standards* (WS/T424-2013).

### 1.5 Statistical analysis

Data were analyzed using SPSS 24.0. Quantitative data were described as means ± SD (normally distributed) or medians (IQR) (non-normally distributed); qualitative data as percentages.

Continuous variables were compared using ANOVA, categorical variables with Pearson’s chi-square or Fisher’s exact test (*p*-values adjusted with Benjamini-Hochberg). Since the outcome variable was binary (pre-frailty vs. frailty), binary logistic regression was used to identify factors associated with frailty status, using the pre-frailty group as the reference (*α* = 0.05). A multi-layer perceptron neural network was subsequently applied as a supplementary analysis to explore the non-linear predictive capacity and variable importance ranking. Missing data were handled using complete case analysis. Participants with missing values for any variable included in a specific model were excluded from that analysis. The rate of missing data for primary variables was less than 2%.

To ensure robust evaluation, the dataset was randomly split into a training set (70%) and a test set (30%). The model was built and tuned exclusively on the training set, and its final performance was evaluated on the untouched test set. For the neural network, a sensitivity analysis was conducted to determine the optimal architecture. We compared models with 1, 3, 5, and 7 hidden layers. A hyperparameter optimization process was conducted using a grid search. We systematically evaluated combinations of hidden layers (1, 3, 5, 7) and node configurations. Model performance was assessed on a validation set (30% of the training data) using the area under the ROC curve (AUC) as the primary metric. The architecture with 7 hidden layers (17 nodes each) was selected as it yielded the highest validation AUC, indicating optimal performance without clear signs of overfitting. The model used the hyperbolic tangent activation function for hidden layers and the Softmax function for the output layer. It was trained using the backpropagation algorithm with a learning rate of 0.01 and the Adam optimizer for 200 epochs.

## 2 Results

### 2.1 General characteristics of pre-frailty and frail older adults

Of 1,451 participants, 784 (54.0%) were pre-frailty, and 667 (46.0%) were frail; of the total sample (*N* = 1,451), 648 (44.7%) were male and 803 (55.3%) were female. Detailed distributions are shown in Table 1.

**Table 1:** General characteristics of pre-frailty and frail community-dwelling older adults.

Variable	Frailty (*N* = 667)	Pre-frailty (*N* = 784)	Statistic	*p* value
Age [mean(SD)]	72.84 (4.99)	70.89 (4.35)	43.740	<0.001
Male	39.88	48.72	11.407	<0.001
Marital status (%)				
Married	84.26	89.16	7.629	0.022
Widowed	14.54	9.95		
Other	1.20	0.89		
Education level (%)				
≤ Primary	47.08	34.78	26.140	<0.001
<3,000 CNY	61.02	51.79	13.911	
≥ College	25.04	35.17		
Urban/Rural (%)				
Urban	37.48	45.66	9.911	0.002
Rural	62.52	54.34		
Monthly income (%)				
<3,000 CNY	61.02	51.79	13.911	
3,000–5,000 CNY	29.54	34.44		
>5,000 CNY	9.45	13.78		
Living condition (%)				
With family	91.84	87.56	7.253	0.009
Alone	8.16	12.44		
Medical insurance (%)				
Yes	98.20	98.72	0.662	0.519
Smoking status (%)				
Current/ex-smoker	25.64	33.72	11.196	<0.001
Never	74.36	66.28		
Alcohol use (%)				
Never	81.71	74.74	10.168	0.001
Chronic diseases (%)				
≤2	78.68	86.48	15.456	<0.001
≥3	21.32	13.52		
Sleep duration [mean(SD)]	6.75 (1.57)	6.82 (1.51)	0.694	0.405

### 2.2 Nutritional risk comparison

In the frailty group, 306 individuals (45.9%) had elevated nutritional risk, compared to 235 (30.0%) in the pre-frailty group (χ^2^ = 38.977, *p* < 0.001). SNAQ indicators (appetite, satiety, taste, meal frequency) differed significantly between groups (Table 2).

**Table 2:** SNAQ indicator distribution in pre-frailty and frailty older adults.

Variable	Percent	Frailty (*N* = 667)	Pre-frailty (*N* = 784)	Statistic	*p* value
Appetite					
Very poor	%	0.15	0.25	79.636	<0.001
Poor	%	11.09	1.66		
Average	%	38.38	33.93		
Good	%	43.78	49.49		
Very good	%	6.60	14.67		
Satiety					
After a few bites	%	4.65	1.91	72.851	<0.001
After 1/3 meal	%	13.19	3.19		
After half meal	%	30.43	27.93		
After full meal	%	49.33	61.74		
Rarely satiated	%	2.40	5.23		
Taste perception					
Poor	%	7.50	0.77	71.819	<0.001
Average	%	38.68	30.48		
Good	%	47.82	55.74		
Very good	%	6.00	13.01		
Meal frequency					
≤1/day	%	2.55	0.77	19.318	0.001
2/day	%	7.95	5.48		
3/day	%	89.20	91.84		
>3/day	%	0.30	1.91		

### 2.3 Sarcopenia prevalence comparison

In the frailty group, 136 individuals (20.4%) had sarcopenia, compared to 44 (5.6%) in the pre-frailty group (Fisher’s exact = 71.952, *p* < 0.001). SARC-F indicators differed significantly (Table 3).

**Table 3:** Sarc-F indicator distribution in pre-frailty and frailty older adults.

Variable	Percent	Frailty (*N* = 667)	Pre-frailty (*N* = 784)	Statistic	*p* value
Strength					
No difficulty	%	55.92	73.09	66.127	<0.001
Some difficulty	%	28.79	22.07		
Major difficulty	%	15.29	4.59		
Assisted walking					
No difficulty	%	76.91	93.75	95.085	<0.001
Some difficulty	%	16.64	5.87		
Major difficulty	%	6.45	0.26		
Rising					
No difficulty	%	78.26	92.22	74.480	<0.001
Some difficulty	%	15.29	7.65		
Major difficulty	%	6.45	0.13		
Stair climbing					
No difficulty	%	42.73	70.92	131.423	<0.001
Some difficulty	%	37.63	23.34		
Major difficulty	%	19.64	5.74		
Falls					
No falls	%	78.71	91.33	48.611	<0.001
1–3 falls	%	17.24	7.78		
≥4 falls	%	4.05	0.89		

### 2.4 ADL comparison

In the frailty group, 379 (56.82%) had no ADL impairment, compared to 606 (77.30%) in the pre-frailty group (*χ*^2^ = 89.551, *p* < 0.001) (Table 4).

**Table 4:** ADL levels in pre-frailty and frailty older adults.

Variable	Percent	Frailty (*N* = 667)	Pre-frailty (*N* = 784)	Statistic	*p* value
No impairment	%	56.82	77.30	89.551	<0.001
Mild impairment	%	38.08	22.58		
Moderate impairment	%	4.65	0.00		
Severe impairment	%	0.30	0.13		
Extreme impairment	%	0.15	0.00		

### 2.5 Multivariate logistic regression of factors associated with frailty Statuss

Variables significant in univariate analysis were included in multivariate logistic regression (pre-frailty = 0, frailty = 1). Age, education, smoking, chronic diseases, SNAQ risk, SARC-F sarcopenia, and ADL disability were independent factors significantly associated with frailty status (*p* < 0.05), with nutritional risk (SNAQ, OR = 2.823) and SARC-F sarcopenia (OR = 1.483) showing among the strongest associations with frailty status. This section presents the primary, clinically actionable results of the study (Table 5).

**Table 5:** Multivariate logistic regression of factors associated with frailty status (*n* = 1,451).

Variables	β	SE	Wald $\chi^{2}$	OR	95%CI	*p* value
Age	−0.051	0.013	15.336	0.951	0.927 ~ 0.975	<0.001
Gender	0.053	0.142	0.142	1.055	0.798 ~ 1.394	0.707
Marital status						
Married	0.279	0.559	0.250	1.322	0.442 ~ 3.951	0.617
Widowed	0.197	0.565	0.122	1.218	0.403 ~ 3.684	0.727
Education						
≤Primary	−0.589	0.206	8.199	0.555	0.371 ~ 0.830	0.004
Middle school	−0.431	0.172	6.232	0.650	0.464 ~ 0.912	0.013
Urban/Rural	−0.073	0.250	0.086	0.930	0.570 ~ 1.516	0.77
Monthly income						
<3,000 CNY	−0.250	0.293	0.728	0.779	0.438 ~ 1.383	0.393
3,000–5,000 CNY	−0.133	0.210	0.400	0.875	0.580 ~ 1.322	0.527
Living condition	0.318	0.256	1.536	1.374	0.831 ~ 2.271	0.215
Smoking	0.330	0.147	5.046	1.390	1.043 ~ 1.853	0.025
Alcohol use	0.115	0.162	0.506	1.122	0.817 ~ 1.540	0.477
Chronic diseases	0.389	0.155	6.268	1.475	1.088 ~ 2.001	0.012
SNAQ risk	1.038	0.208	24.976	2.823	1.879 ~ 4.241	<0.001
SARC-F sarcopenia	0.394	0.121	10.657	1.483	1.170 ~ 1.878	0.001
ADL disability	0.435	0.135	10.322	1.545	1.185 ~ 2.014	0.001

### 2.6 Physical measurement comparison and logistic regression

Of 1,425 participants with measurements, frailty group BMI, waist circumference, and systolic blood pressure were higher, while grip strength was lower (*p* < 0.05) (Table 6). Logistic regression confirmed waist circumference, grip strength, and systolic blood pressure as independently associated with frailty status (*p* < 0.05) (Table 7).

**Table 6:** Physical measurements in pre-frailty and frailty older adults (Mean ± SD).

Variables	Unit	Frailty	Pre-frailty	F	*p* value
BMI	Kg/m^2^	25.94 (4.01)	25.51 (3.86)	4.293	0.038
Waist circumference	cm	92.56 (10.82)	90.97 (10.93)	7.596	0.006
Arm circumference	cm	29.05 (5.38)	29.29 (5.39)	0.699	0.403
Calf circumference	cm	34.70 (4.02)	34.81 (4.26)	0.247	0.619
Grip strength	Kgf	22.11 (8.73)	24.64 (8.70)	29.988	<0.001
Triceps skinfold	mm	19.94 (8.30)	19.97 (9.16)	0.003	0.957
Subscapular skinfold	mm	22.10 (7.20)	22.60 (7.61)	1.606	0.205
Systolic BP	mmHg	141 (20)	136 (20)	18.601	<0.001
Diastolic BP	mmHg	77 (11)	76 (12)	4.552	0.033

**Table 7:** Multivariate logistic regression of physical measurements associated with frailty status (*n* = 1,425).

Physical measurements	β	SE	Wald $\chi^{2}$	OR	95%CI	*p* value
Waist Circumference	0.019	0.007	6.742	1.019	1.005 ~ 1.034	0.009
Grip Strength	−0.035	0.007	28.178	0.966	0.953 ~ 0.978	<0.001
Systolic BP	0.009	0.003	6.841	1.009	1.002 ~ 1.015	0.009
Diastolic BP	0.001	0.006	0.06	1.001	0.99 ~ 1.013	0.807
BMI	−0.018	0.02	0.864	0.982	0.945 ~ 1.021	0.353

### 2.7 Neural network prediction

As an exploratory analysis, a multi-layer perceptron neural network was used to provide a complementary perspective on variable importance. The model’s performance on the test set was as follows: overall accuracy 65.2%, precision 88.6%, recall 64.4%, F1-score 0.746, and an area under the ROC curve (AUC) of 0.695.

A multi-layer perceptron neural network analyzed significant variables from logistic regression, with 11 input variables, “frailty status” as the output, and the optimized 7 hidden layers with 17 nodes each using backpropagation, hyperbolic tangent (hidden layer), and Softmax (output layer) functions. Key factors ranked by normalized importance were: SARC-F sarcopenia (100.0%), waist circumference (67.0%), age (62.8%), education (58.1%), ADL disability (55.1%), chronic diseases (49.9%), SNAQ risk (37.0%), smoking (33.9%), BMI (28.8%), systolic blood pressure (19.4%), and grip strength (18.0%). The neural network analysis identified the relative importance of various factors in distinguishing between pre-frail and frail status at the time of assessment. The rankings should be interpreted as the model’s estimate of each variable’s contributory importance within the non-linear framework, and not as effect sizes for clinical use.

For comparison, the logistic regression model applied to the same test set achieved an AUC of 0.648.

## 3 Discussion

This cross-sectional study identified several factors strongly associated with frailty status among community-dwelling older adults, Risk factors for pre-frailty and frailty overlap significantly, including comorbidities, polypharmacy, and malnutrition, which, if unaddressed in pre-frailty, are strongly linked to the frailty state (26). Early detection of changes in physical status and function, followed by interventions such as nutritional support, exercise, and cognitive training, can effectively delay or reverse frailty, improving quality of life and reducing healthcare burden (27, 28).

This study found waist circumference to be a prominent factor associated with frailty status in the neural network model, with related indicator BMI ranking fourth. Compared to BMI, waist circumference better reflects metabolic status and health risks in the older adults. This can be mechanistically explained by the role of visceral adipose tissue, which is metabolically active and a key source of pro-inflammatory cytokines (e.g., IL-6, TNF-*α*) and adipokines. These factors can directly contribute to the pathogenesis of frailty by promoting sarcopenia, insulin resistance, and chronic low-grade inflammation (29). Thus, our finding suggests that the metabolic dysfunction associated with abdominal obesity, rather than overall body mass per se, is a critical pathway in the frailty process. Liu et al. reported a stronger association between waist circumference and frailty than BMI (30), affecting pre-frailty not only directly but also indirectly via metabolic disorders, vitamin D levels, and inflammation. Liao et al.’s study of 6,320 older individuals in Beijing found that abdominal obesity predicted frailty more effectively than general obesity, highlighting waist circumference’s strong association with frailty (31). A study on Chinese older adults showed a positive correlation between weight-adjusted waist index and frailty, emphasizing central obesity’s role (32). Thus, regular waist circumference monitoring may be a valuable indicator for identifying older adults with pre-frailty who are at a cross-sectional level of higher risk. It directly reflects abdominal and visceral fat accumulation, closely tied to metabolic disease risk, whereas BMI only indicates overall weight-to-height ratio without distinguishing fat distribution. Being simple to measure, waist circumference should be prioritized in frailty prevention, particularly for pre-frail older adults, and combined with BMI for comprehensive assessment.

ADL disability, assessed via the MBI, was another key risk factor. Lower MBI scores are strongly associated with frailty status compared to pre-frailty, consistent with its ability to quantify daily living capacity and detect early risk (33). Timely assessment and intervention by community workers or families may help mitigate frailty.

Age was also a significant risk factor, with older age being strongly associated with frailty status, aligning with prior studies. As age advances, organ degeneration and physiological decline reduce resilience.

Systolic blood pressure was an independent risk factor, higher in the frailty group (141 vs. 136 mmHg). However, findings on systolic blood pressure are inconsistent. A Korean study of 2,697 older individuals found frailty prevalence rates of 39.2 and 44.2% in pre-frail and frail groups, respectively (34). Odden et al. linked low systolic blood pressure to poor outcomes in frail patients (35), Matsuoka et al. found a negative correlation (36), while a domestic study found no significant association after adjusting confounders (37). Tan et al.’s study of 260 hypertensive community patients reported a 31.37% frailty rate, suggesting a bidirectional relationship (38). Further research is needed to clarify mechanisms. Nonetheless, regular systolic blood pressure monitoring and interventions (lifestyle adjustments, medication) are recommended to maintain optimal levels and reduce frailty risk.

Grip strength, an indicator of muscle strength, was significantly lower in the frailty group. A cohort study found lower grip strength in middle-aged and older individuals was associated with frailty (39). Reduced grip strength reflects muscle loss and is linked to overall functional decline and quality of life, with chronic inflammation and metabolic disorders contributing to muscle dysfunction (40).

Both analytical models converged in highlighting the importance of muscular and functional factors. SARC-F sarcopenia was identified as a key correlate by logistic regression and was assigned the highest normalized importance by the neural network. This consistency across methodologies strengthens the evidence for sarcopenia as a central component of the frailty phenotype in our cross-sectional sample. The neural network further suggested strong non-linear contributions from waist circumference and age, complementing the linear associations quantified by logistic regression. This alignment between a traditional interpretable model and a complex machine learning approach reinforces the robustness of the identified core factors.

### 3.1 Comparison of modeling approaches

This study employed two complementary modeling strategies. The logistic regression model provides the core, interpretable findings for clinical practice, clearly quantifying how each factor (e.g., SARC-F sarcopenia, ADL disability) is associated with frailty status through Odds Ratios. In contrast, the neural network, while demonstrating high capability to distinguish between groups, functions as a “black box.” Its value in this context lies in its ability to validate the complex, non-linear nature of frailty and to offer a different perspective on variable importance, which largely aligned with the logistic regression results. For the purpose of developing actionable screening guidelines or interventions, the outputs of the logistic regression model—which identified nutritional risk (SNAQ), sarcopenia (SARC-F), and ADL disability as significant factors—are inherently more useful and should form the basis of decision-making.

### 3.2 Study limitations and strengths

When interpreting the findings of this cross-sectional study, several important limitations must be acknowledged, which also define the scope and appropriate application of our results.

First and foremost, the cross-sectional design fundamentally limits the inference of causality. Although we identify factors associated with frailty status, we cannot establish temporal sequence or determine whether these associated factors are causes or consequences of the frailty state. The predictive performance of our models reflects their ability to classify current status cross-sectionally, not to forecast future events.

Second, despite including a range of variables, our study lacked data on several important potential confounders, such as psychosocial factors (e.g., social isolation, depression), cognitive function, detailed medication use (polypharmacy), objective physical activity levels, and environmental influences. The absence of these variables increases the risk of residual confounding, and our model should be viewed as identifying core associative factors rather than exhaustive causal determinants.

Third, the generalizability of our findings is constrained by the use of a convenience sampling method from specific districts in Tianjin, which may not be representative of the broader older population in China or other cultural contexts.

Fourth, regarding measurement, although all investigators underwent standardized training, we did not quantitatively assess inter-rater reliability for the questionnaires or physical measurements, which is a limitation for assessing measurement consistency. Furthermore, frailty was classified solely using the FRAIL scale. While practical for community screening, this choice may limit the direct comparability of our findings with studies employing alternative, multi-domain frailty criteria like the Fried phenotypic model or the Frailty Index.

Finally, concerning the analytical approach, the neural network model, while valuable for capturing complex patterns, was not externally validated, and its inherent lack of interpretability limits its direct clinical utility compared to the logistic regression model.

Despite these limitations, our study also has notable strengths, including a relatively large sample size, the simultaneous assessment of a wide array of biomedical and functional risk factors, and the use of both traditional and machine-learning approaches to provide complementary insights.

## 4 Conclusion

In conclusion, this cross-sectional study identified several factors strongly associated with frailty status in community-dwelling older adults. Among these, nutritional risk (SNAQ), SARC-F sarcopenia, and ADL disability emerged as significant clinically interpretable markers. The complementary neural network analysis affirmed the multifactorial nature of frailty and underscored the paramount importance of sarcopenia within the complex risk profile.

For immediate clinical and public health practice, our findings suggest that simple, low-cost tools like the SNAQ, SARC-F questionnaire, and ADL scale should be prioritized in community settings to identify high-risk individuals efficiently.

While these cross-sectionally identified factors represent promising targets for screening and early intervention, their role in the progression from pre-frailty to frailty must be investigated through future longitudinal studies. Such research will be essential to confirm the cost-effectiveness of implementing these assessments on a broader scale and to establish evidence-based, preventive healthcare strategies for the aging population.

## Data Availability

The original contributions presented in the study are included in the article/Supplementary material, further inquiries can be directed to the corresponding author.
