# Supplementary material for: Factors associated with frailty status compared to pre-frailty in community-dwelling older adults: a cross-sectional study
Source: Front Public Health. 2026 Jan 14;13:1728208. doi: 10.3389/fpubh.2025.1728208 (PMC12846931; doi:10.3389/fpubh.2025.1728208)
Supplement: Supplementary file 1 [file Table_1.docx]

Supplementary 1 Questionnaire for Pre-Frailty and Frailty Elderly Individuals

District Name: __________ {Dis}

District Code: __________ {DisID}

Personal Code: __________ {PID}

Personal Code (2015): __________ {PID2015}

I. Basic Information

1.Name: __________ {name}

2.Gender: {sex} ① Male ② Female

3.ID Number: __________ {IDcard}

4.Phone Number: __________ {tel}

5.Date of Birth: <yyyy/mm/dd> {birthdate}

6.Home Address: __________ {address}

7.Height: #.## meters {height}

8.Weight: ###.# kg {weight}

9.Marital Status: {A9} ① Unmarried ② Married ③ Divorced ④ Widowed

10.Educational Level: {A10} ① Elementary School or Below ② Junior High School ③ Senior High School/Vocational School/Secondary Technical School ④ College/University Bachelor's Degree ⑤ Master's Degree or Above

11.Occupation Before Retirement: {A11} ① Party, Government, Mass Organizations, Social Organizations, Enterprise, and Public Institution Leaders, Civil Servants ② Company Staff ③ Private Enterprise Owners ④ Professionals (Teachers, Doctors, Researchers, Engineers, Judges, etc.) ⑤ Self-Employed Individuals ⑥ Commercial and Service Industry Employees ⑦ Industrial Workers ⑧ Agricultural Workers ⑨ Unemployed/U就业less ⑩ Military Personnel ⑪ Housekeeping and Other

12.Your Total Monthly Income: {A12} ① Below 1000 CNY ② 1001-2999 CNY ③ 3000-5000 CNY ④ Above 5000 CNY

13.Number of Children: ## {A13}

15.Current Living Arrangement: {A14} ① Living with Spouse and Children ② Living Only with Spouse ③ Living Only with Children ④ Living Alone ⑤ Other

15.Can You Afford Daily Medical Expenses? (Excluding Major Illnesses): {A15} ① Yes ② Basically Yes ③ Some Difficulty ④ No

16.Do You Have Social Medical Insurance, Public-funded Medical Care, or Cooperative Medical Care?: {A16} ① Yes ② No

II. Physical Condition

17.Overall, How Do You Rate Your Physical Condition?: {A17} ① Very Good ② Good ③ Fair ④ Poor ⑤ Very Poor

18.How Many Times Have You Seen a Doctor This Year?: {A18} ① Never Went ② Less Than 5 Times ③ 5-10 Times ④ More Than 10 Times

19.Did You Have a Physical Examination at the Hospital This Year?: {A19} ① Yes ② No

20.Is It Convenient for You to Seek Medical Care at the Hospital?: {A20} ① Convenient (Jump to Question 22) ② Fairly Convenient (Jump to Question 22) ③ Average (Jump to Question 22) ④ Fairly Inconvenient ⑤ Inconvenient

21.If Inconvenient, What Are the Reasons? (Multiple Choices)

21a1.Inconvenient Transportation {A21a1} ① Yes ② No

21a2.No One to Accompany {A21a2} ① Yes ② No

21a3.Difficulty in Seeking Medical Care {A21a3} ① Yes ② No

21a4.Unclear Which Department to Visit {A21a4} ① Yes ② No

21a5.Mobility Issues {A21a5} ① Yes ② No

21a6.Long Distance {A21a6} ① Yes ② No

21a7.Other (Please Specify): __________ {A21a7}

22.Do You Suffer from Any of the Following Diseases? (Multiple Choices)

22a1.Hypertension {A22a1} ① Yes ② No

22a2.Heart Disease {A22a2} ① Yes ② No

22a3.Malignant Tumor {A22a3} ① Yes ② No

22a4.Diabetes {A22a4} ① Yes ② No

22a5.Lung Disease {A22a5} ① Yes ② No

22a6.Abnormal Blood Lipids {A22a6} ① Yes ② No

22a7.Eye Disease {A22a7} ① Yes ② No

22a8.Physical Disability {A22a8} ① Yes ② No

22a9.Stroke {A22a9} ① Yes ② No

22a10.Cognitive Impairment (Dementia) {A22a10} ① Yes ② No

22a11.Mental Disorder {A22a11} ① Yes ② No

22a12.Parkinson's Disease {A22a12} ① Yes ② No

22a13.Other (Please Specify): __________ {A22a13}

23.Do You Experience Any of the Following Symptoms? (Multiple Choices)

23a1.Memory Loss {A23a1} ① Yes ② No

23a2.Mental Confusion {A23a2} ① Yes ② No

23a3.Visual Impairment {A23a3} ① Yes ② No

23a4.Hearing Impairment {A23a4} ① Yes ② No

23a5.Difficulty Walking {A23a5} ① Yes ② No

23a6.Difficulty Eating {A23a6} ① Yes ② No

23a7.Other Health Issues (Please Specify): __________ {A23a7}

24.In the Past Three Months, Have You Had Difficulty Performing Any of the Following Activities? (Multiple Choices)

24a1.Eating {A24a1} ① Yes ② No

24a2.Bathing {A24a2} ① Yes ② No

24a3.Getting In/Out of Bed {A24a3} ① Yes ② No

24a4.Dressing {A24a4} ① Yes ② No

24a5.Cleaning the House {A24a5} ① Yes ② No

24a6.Walking Indoors {A24a6} ① Yes ② No

24a7.Using the Toilet {A24a7} ① Yes ② No

24a8.Buying Groceries {A24a8} ① Yes ② No

III. Diet and Lifestyle

25.How Many Hours of Sleep Do You Get Daily?: {A25} ## hours

26.Do You Currently Smoke?: {A26} ① Currently Smoke ② Have Quit Smoking ③ Never Smoked

27.What Is Your Current Alcohol Consumption?: {A27} ① Drank Beer, Spirits, or Other Alcoholic Beverages in the Past Year ② Never Drink

28.Do You Exercise Regularly?: {A28} ① No Exercise ② 1-3 Times per Week ③ 4 Times or More per Week

29.What Is Your Breakfast Habit?: {A29} ① Eat Every Day ② Do Not Eat Every Day

30.Do You Drink Water Before Breakfast?: {A30} ① Yes ② No

31.Do You Have a Snack After Dinner?: {A31} ① Yes ② No

32.What Is Your Current Dietary Taste Preference?: {A32} ① Light ② Moderate ③ Heavy

33.How Much Water Do You Drink Daily?: {A33} ① 1.5 Liters or More ② Less than 1.5 Liters

34.What Time Do You Have Lunch?: {A34} ① Before 12 PM ② After 12 PM

35.What Is Your Current Daily Fat Intake?: {A35} ① ≤25g/d ② 25-35g/d ③ >35g/d

36.Compared to 10 Years Ago, How Has Your Dietary Taste Changed?: {A36} ① Became Saltier ② Became Lighter ③ No Significant Change
