# Supplementary material for: Factors associated with frailty status compared to pre-frailty in community-dwelling older adults: a cross-sectional study
Source: Front Public Health. 2026 Jan 14;13:1728208. doi: 10.3389/fpubh.2025.1728208 (PMC12846931; doi:10.3389/fpubh.2025.1728208)
Supplement: Supplementary file 2 [file Table_2.docx]

Supplement Table 1. Multivariate Logistic Regression of Frailty Risk Factors (Male)

| **Risk factors** | **β** | **SE** | **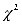Wald** | **OR** | **95%CI** | ***P*** Value |
| --- | --- | --- | --- | --- | --- | --- |
| Age | -0.049 | 0.019 | 6.334 | 0.952 | 0.917~0.989 | 0.012 |
| Marital Status |  |  |  |  |  |  |
| Married | 0.023 | 0.996 | 0.001 | 1.024 | 0.145~7.208 | 0.981 |
| Widowed | 0.932 | 1.009 | 0.853 | 2.540 | 0.351~18.358 | 0.356 |
| Education |  |  |  |  |  |  |
| ≤Primary | -0.514 | 0.303 | 2.872 | 0.598 | 0.330~1.084 | 0.090 |
| Middle School | -0.379 | 0.261 | 2.109 | 0.684 | 0.410~1.142 | 0.146 |
| Urban/Rural | -0.328 | 0.347 | 0.895 | 0.720 | 0.365~1.421 | 0.344 |
| Monthly Income |  |  |  |  |  |  |
| <3000 CNY | -0.227 | 0.402 | 0.319 | 0.797 | 0.362~1.752 | 0.572 |
| 3000-5000 CNY | -0.06 | 0.300 | 0.040 | 0.942 | 0.523~1.695 | 0.841 |
| Living Condition | 1.113 | 0.516 | 4.642 | 3.043 | 1.106~8.373 | 0.031 |
| Smoking | 0.279 | 0.183 | 2.313 | 1.321 | 0.923~1.892 | 0.128 |
| Alcohol Use | 0.036 | 0.180 | 0.041 | 1.037 | 0.729~1.475 | 0.840 |
| Chronic Diseases | 0.555 | 0.238 | 5.435 | 1.742 | 1.092~2.778 | 0.020 |
| SNAQ Risk | 0.425 | 0.184 | 5.329 | 1.529 | 1.066~2.192 | 0.021 |
| SARC-F Sarcopenia | 0.924 | 0.346 | 7.139 | 2.520 | 1.279~4.963 | 0.008 |
| ADL Disability | 0.516 | 0.207 | 6.225 | 1.676 | 1.117~2.514 | 0.013 |

Supplement Table 2. Multivariate Logistic Regression of Frailty Risk Factors (Female)

| **Risk factors** | **β** | **SE** | **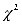Wald** | **OR** | **95%CI** | ***P*** Value |
| --- | --- | --- | --- | --- | --- | --- |
| Age | -0.058 | 0.018 | 10.541 | 0.943 | 0.911~0.977 | 0.001 |
| Marital Status |  |  |  |  |  |  |
| Married | 0.421 | 0.689 | 0.374 | 1.524 | 0.395~5.880 | 0.541 |
| Widowed | 0.010 | 0.699 | 0 | 1.010 | 0.257~3.975 | 0.989 |
| Education |  |  |  |  |  |  |
| ≤Primary | -0.606 | 0.291 | 4.350 | 0.545 | 0.309~0.964 | 0.037 |
| Middle School | -0.492 | 0.233 | 4.467 | 0.612 | 0.388~0.965 | 0.035 |
| Urban/Rural | 0.271 | 0.371 | 0.534 | 1.311 | 0.634~2.712 | 0.465 |
| Monthly Income |  |  |  |  |  |  |
| <3000 CNY | -0.216 | 0.438 | 0.242 | 0.806 | 0.341~1.903 | 0.622 |
| 3000-5000 CNY | -0.261 | 0.299 | 0.760 | 0.771 | 0.429~1.384 | 0.383 |
| Living Condition | 0.093 | 0.306 | 0.093 | 1.098 | 0.602~2.000 | 0.761 |
| Smoking | 0.43 | 0.254 | 2.862 | 1.537 | 0.934~2.529 | 0.091 |
| Alcohol Use | 0.479 | 0.407 | 1.381 | 1.614 | 0.726~3.586 | 0.240 |
| Chronic Diseases | 0.243 | 0.207 | 1.380 | 1.276 | 0.850~1.915 | 0.240 |
| SNAQ Risk | 0.417 | 0.163 | 6.585 | 1.517 | 1.104~2.087 | 0.010 |
| SARC-F Sarcopenia | 1.110 | 0.264 | 17.696 | 3.036 | 1.810~5.093 | <0.001 |
| ADL Disability | 0.384 | 0.181 | 4.487 | 1.468 | 1.029~2.093 | 0.034 |

Supplement Table 3. Multivariate Logistic Regression of Frailty Risk Factors (Age<70)

| **Risk factors** | **β** | **SE** | **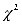Wald** | **OR** | **95%CI** | ***P*** Value |
| --- | --- | --- | --- | --- | --- | --- |
| Gender | -0.018 | 0.067 | 0.075 | 0.982 | 0.861~1.119 | 0.784 |
| Marital Status | -0.049 | 0.246 | 0.04 | 0.952 | 0.588~1.543 | 0.842 |
| Married |  |  |  |  |  |  |
| Widowed | 1.286 | 0.917 | 1.966 | 3.618 | 0.599~21.840 | 0.161 |
| Education | 1.596 | 0.921 | 3.003 | 4.931 | 0.811~29.966 | 0.083 |
| ≤Primary |  |  |  |  |  |  |
| Middle School | -0.566 | 0.316 | 3.214 | 0.568 | 0.306~1.054 | 0.073 |
| Urban/Rural | -0.332 | 0.267 | 1.545 | 0.717 | 0.425~1.211 | 0.214 |
| Monthly Income | 0.442 | 0.412 | 1.149 | 1.555 | 0.693~3.489 | 0.284 |
| <3000 CNY |  |  |  |  |  |  |
| 3000-5000 CNY | 0.444 | 0.496 | 0.803 | 1.559 | 0.590~4.120 | 0.370 |
| Living Condition | 0.037 | 0.344 | 0.012 | 1.038 | 0.528~2.038 | 0.914 |
| Smoking | 0.506 | 0.497 | 1.035 | 1.659 | 0.626~4.397 | 0.309 |
| Alcohol Use | 0.255 | 0.254 | 1.012 | 1.291 | 0.782~2.123 | 0.314 |
| Chronic Diseases | 0.126 | 0.272 | 0.214 | 1.134 | 0.666~1.931 | 0.644 |
| SNAQ Risk | 0.494 | 0.268 | 3.394 | 1.638 | 0.969~2.770 | 0.065 |
| SARC-F Sarcopenia | 0.226 | 0.207 | 1.189 | 1.254 | 0.835~1.882 | 0.276 |
| ADL Disability | 1.387 | 0.374 | 13.738 | 4.001 | 1.922~8.329 | <0.001 |

Supplement Table 4. Multivariate Logistic Regression of Frailty Risk Factors (Age≥70)

| **Risk factors** | **β** | **SE** | **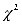Wald** | **OR** | **95%CI** | ***P*** Value |
| --- | --- | --- | --- | --- | --- | --- |
| Gender | -0.044 | 0.020 | 4.870 | 0.957 | 0.921~0.995 | 0.027 |
| Marital Status | 0.151 | 0.177 | 0.731 | 1.163 | 0.822~1.646 | 0.393 |
| Married |  |  |  |  |  |  |
| Widowed | -0.928 | 0.877 | 1.117 | 0.396 | 0.071~2.208 | 0.290 |
| Education | -1.182 | 0.899 | 1.727 | 0.307 | 0.053~1.787 | 0.189 |
| ≤Primary |  |  |  |  |  |  |
| Middle School | -0.574 | 0.281 | 4.176 | 0.563 | 0.325~0.977 | 0.041 |
| Urban/Rural | -0.510 | 0.235 | 4.721 | 0.601 | 0.379~0.951 | 0.030 |
| Monthly Income | -0.334 | 0.326 | 1.049 | 0.716 | 0.378~1.357 | 0.306 |
| <3000 CNY |  |  |  |  |  |  |
| 3000-5000 CNY | -0.646 | 0.373 | 3.004 | 0.524 | 0.253~1.088 | 0.083 |
| Living Condition | -0.217 | 0.270 | 0.644 | 0.805 | 0.474~1.367 | 0.422 |
| Smoking | 0.136 | 0.310 | 0.193 | 1.146 | 0.624~2.106 | 0.660 |
| Alcohol Use | 0.395 | 0.183 | 4.682 | 1.485 | 1.038~2.125 | 0.030 |
| Chronic Diseases | 0.089 | 0.203 | 0.191 | 1.093 | 0.734~1.628 | 0.662 |
| SNAQ Risk | 0.322 | 0.195 | 2.727 | 1.380 | 0.942~2.021 | 0.099 |
| SARC-F Sarcopenia | 0.527 | 0.152 | 11.94 | 1.693 | 1.256~2.283 | 0.001 |
| ADL Disability | 0.860 | 0.252 | 11.66 | 2.363 | 1.442~3.871 | 0.001 |
